# The Ameliorative Effect of Mahuang Fuzi and Shenzhuo Decoction on Membranous Nephropathy of Rodent Model is Associated With Autophagy and Wnt/β-Catenin Pathway

**DOI:** 10.3389/fphar.2022.820130

**Published:** 2022-04-21

**Authors:** Yu Gao, Haoran Dai, Na Zhang, Hanxue Jiang, Zihan Zhang, Zhendong Feng, Zhaocheng Dong, Wenbin Liu, Fei Liu, Xuan Dong, Qihan Zhao, Xiaoshan Zhou, Jieli Du, Naiqian Zhang, Hongliang Rui, Baoli Liu

**Affiliations:** ^1^ Beijing Hospital of Traditional Chinese Medicine, Capital Medical University, Beijing, China; ^2^ School of Traditional Chinese Medicine, Capital Medical University, Beijing, China; ^3^ Shunyi Branch, Beijing Hospital of Traditional Chinese Medicine, Beijing, China; ^4^ School of Life Sciences, Beijing University of Chinese Medicine, Beijing, China; ^5^ Pinggu Hospital, Beijing Hospital of Traditional Chinese Medicine, Beijing, China; ^6^ Beijing Institute of Chinese Medicine, Beijing, China

**Keywords:** mahuang fuzi and shenzhuo decoction, podocyte injury, autophagy, wnt/β-catenin, membranous nephropathy

## Abstract

The increased incidence of membranous nephropathy (MN) has made it the most common pathological type of primary nephrotic syndrome in adults in China. According to the theory of Traditional Chinese Medicine (TCM), Mahuang Fuzi (Chinese ephedra and Radix Aconiti Lateralis Preparata) and Shenzhuo Decoction (MFSD) could be used to treat such diseases. We treated patients of MN with MFSD, and observed comparable efficacy to glucocorticoid and/or immunosuppressants. In this study, we observed the therapeutic effect of MFSD on the rat model of passive Heymann nephritis (PHN), a classical MN model. Our results showed that MFSD treatment significantly reduced urinary protein level and podocyte injury in PHN rats, and correspondingly improved renal pathology, with the improvement effect on MN comparable to that of Cyclosporine A (CsA) alone. To explore the potential therapeutical mechanism of MFSD, the main chemical components of MFSD were determined by High-performance liquid chromatography-mass spectrometry (HPLC-MS). There were about 30 active components of MFSD. Next, based on network pharmacology methods, we screened related targets of MSFD on MN, which provided a preliminary understanding of the MFSD bioactive compounds. The clustering analysis showed that its active site might be in the autophagy-related protein and Wnt/β-catenin pathway, which was related to podocyte injury. Finally, we observed an improvement in renal autophagy and a down-regulation of the Wnt/β-catenin pathway after MSFD treatment in a PHN rat model. According to this study, autophagy and Wnt/β-catenin pathway may be potential targets for MFSD in the treatment of MN.

## Introduction

Membranous nephropathy (MN) is an immune-mediated glomerular disease whose pathological characteristics are the immune deposits under the glomerular capillary epithelium and thereby the diffuse thickening of the basement membrane. It is a major cause of non-diabetic nephrotic syndrome (Liu et al., 2019). In China, the incidence of MN has been increasing year by year, and the detection rate of MN in elderly patients over 60 years old with nephrotic syndrome was 67. 3% (Xu et al., 2016; Xiong et al., 2020). At present, the common background medications for MN are glucocorticoids, cyclophosphamide, calcineurin inhibitors (CNIs), and rituximab. However, high recurrence rate and low complete remission rate still exist, and the side effects such as infection caused by immunosuppression need to be overcome (von Mering et al., 2003; Qiu et al., 2017). The herbs can effectively improve membranous nephropathy (Ahmed et al., 2007; Liu et al., 2019; Tian et al., 2019) *via* this multi-target treatment of membranous nephropathy. Herein, it is possible to treat MN by repairing the function of podocytes (Feng et al., 2020).

Network pharmacology is a developing paradigm that uses multi-disciplinary technologies such as system biology, multi-directional pharmacology, computational biology, and network analysis to expose the fundamental molecular targets and pharmacodynamics methods of TCM in a systematic manner (Hopkins, 2008; Li and Zhang, 2013). Using multiple technologies, such as Omics technology, high-throughput screening, network visualization, or network analysis, the multi-level association between “drug-compound-target pathway-disease” was established using this approach. Understanding the molecular foundation of disease, predicting the pharmacological mechanism, and identifying herbal substances with high efficacy and minimal toxicity were all provided as inspirations.

Mahuang Fuzi and Shenzhuo Decoction (MFSD), as a classic prescription in Treatise on Febrile Diseases used clinically for 1800 years, consists of 6 kinds of Chinese herbs, namely ephedra, aconite, dried ginger, poria cotta, atractylodes, and licorice. These ingredients have been reported in the literature for kidney disease treatment (Tan et al., 2014; Liu et al., 2019; Dai et al., 2020). MFSD activates podocyte autophagy by inhibiting activation of the Wnt/β-catenin signaling pathway and alleviates hyperglycemia-induced podocyte injury (Dai et al., 2020). According to the TCM theory, MFSD can treat MN through warming yang and resolving exterior methods. No study has been conducted on the treatment of membranous nephropathy with MFSD. However, in our previous clinical trials, the efficacy of MFSD in the treatment of membranous nephropathy has been observed, but the mechanism is still unclear (Dong et al., 2021).

The Wnt/β-catenin pathway influences different nephritic-related diseases, renal fibrosis, acute renal failure, and ischemic injury (Kawakami et al., 2013). Activation of the Wnt/β-catenin pathway has been found in the glomeruli of both FSGS and diabetic nephropathy patients, damaging podocytes and leading to proteinuria formation (Dai et al., 2009; Kato et al., 2011). Activation of the Wnt/β-catenin pathway and inhibition of autophagy could also be observed in high-glycemic podocytes (Dai et al., 2020). Insufficient autophagy of podocytes can be observed in podocyte disease (Liu et al., 2017; Jin et al., 2018; Li et al., 2021). According to previous studies, C5b-9 blocks the lysosome-dependent autophagy pathway, leading to podocyte injury (Liu et al., 2017). It can be seen that podocyte autophagy is down-regulated in MN. Autophagy is also regulated by various mechanisms, including the Wnt/β-catenin pathway (Petherick et al., 2013; Kühn et al., 2015; Zhou et al., 2019; Yun et al., 2020). In our previous studies, changes in the Wnt/β-catenin pathway and autophagy in podocytes incubated with C5b-9 were observed, but it was unclear whether MFSD could improve MN through the Wnt/β-catenin pathway and regulate autophagy.

In this study, passive Heymann nephritis (PHN) model was used to observe the therapeutic effect of MFSD on MN rodent model, and a deliberate strategy integrating network pharmacology and pharmacodynamics methods was employed to investigate the potential target of MFSD for the MN treatment.

## Materials and Methods

### Animals and Model Establishment

Sprague-Dawley (SD) rats in special pathogen-free (SPF) levels were fed adaptively for 3 days before animal experiments. Beijing Huafukang Biotechnology Co., Ltd. provided the rats (No. SCXK Jing 2016–0,002) and housed them at the Beijing Institute of Chinese medicine. All experiments were conducted following the internationally recognized standard guidelines for using animals and were reviewed and approved by the Experimental Animal Welfare Ethics Committee of the Beijing Institute of Chinese Medicine (Ethics No. 2019060103). Thirty-two male SD rats weighing between 150 and 180 g were raised under standard environmental conditions (21 ± 2°C, 55 ± 5% humidity, 12 h/12 h light/dark cycle in SPF condition) and offered with free water and standard laboratory diet.

Eight rats in the normal group were injected intravenously with normal saline 0.5 ml/100g, while the rest were intravenously injected with anti-Fx1A Serum (Probetex, Beijing, China) 0.5 ml/100 g once. After 1 week, the 24 h urine proteinuria was detected, proving the success of the model. Then, the successful model rats were further randomly divided into three groups: model group, MFSD group, and Cyclosporine A (CsA, Huadong Medicine Co., Ltd., Hangzhou, China) group. All treatment groups were treated for 12 weeks by giving medicine by gavage every day. The dose of MFSD was 1 ml/100 g/d, and CsA of 25 mg/kg/d. Since CsA was the main therapeutic regimen for MN (Floege et al., 2019), we chose CsA as the positive control drug. The dose was selected according to the equivalent human dose in clinical use. The normal and model groups received the same volume of distilled water.

### Urine and Serum Collection and Biochemical Analysis

After the final treatment, the 24-h urine was collected in a metabolic cage, and the urine protein was quantified by the Laboratory Department of Beijing Hospital of Traditional Chinese Medicine, Capital Medical University. Rats were anesthetized with 1% sodium pentobarbital before obtaining their blood from the abdominal aorta. After centrifugation, the serum was stored at -80°C until analysis, and the serum biochemical indexes (ALB, CHO, TG, BUN, Cr, ALT, AST) were measured with Hitachi 7,600 automatic biochemical analyzer.

### Histological Analysis of Renal Tissues

The kidney tissues were fixed with 4% paraformaldehyde. After 24 h, the tissue was embedded in paraffin, cut into 4 μm thick sections, and stained with hematoxylin and eosin (HE), periodic acid-silver methenamine (PASM), and Masson according to standard protocols.

In order to evaluate the severity of tubulointerstitial injury, semi-quantitative renal tubule-interstitium score were used as described (Li et al., 2012). Briefly, the tubular atrophy, interstitial inflammation, and fibrosis area were evaluated in 10 fields on each HE stained renal section under a microscope with ×100 magnification. The score was graded from 0 to 3 points (0 points, normal tubulointerstitial; 1 point, lesion area <25% of the section; 2 points, lesion area in 25–50% of the section; and 3 points, lesion area >50% of the section). The mean value was referred to as the tubulointerstitial lesion index.

### Immunohistochemical Assay

Kidney tissues were fixed with 4% paraformaldehyde, embedded in paraffin, and cut into 3 mum-thick sections. For antigen retrieval, the sections were placed in 10 mm sodium citrate buffer (pH 6. 0) and heated to near boiling (95–98°C) in a water bath for 20 min (or an oven), followed by 20 min cooling at room temperature. The antigen was repaired with sodium citrate and then immersed in 3% H_2_O_2_ for 15 min to remove endogenous catalase.

Afterward, the sections were added to goat serum blocking solution at room temperature for 30 min and then incubated with rabbit anti-GSK-3β (#27C10, CST, United States) overnight at 4°C. Next the sections were incubated with an HRP-labeled secondary antibody (#SP-00223, Bioss, Beijing). Finally, DAB was added and stained with hematoxylin. All sections were taken under a light microscope with a 400× (Zeiss Axio Imager, Germany). Brown color would be considered as positive staining.

### Immunofluorescence Assays

Kidney tissues were fixed with 4% paraformaldehyde, embedded in paraffin, and cut into 3 mum-thick sections. After dewaxing and hydration, sodium citrate buffer was added before heating in a water bath and cooling to room temperature. The sections were incubated with rabbit anti-LC3A/B (#12741, CST, United States), rabbit anti-p62 (#16177, CST, United States), rabbit anti-β-catenin (#8480, CST, United States) or rabbit anti-nephrin (#ab216341, Abcam, UK) respectively at 4°C overnight. Afterward, the sections were added with Alexa Fluor 488 (Thermo Fisher Scientific, United States) or Alexa Fluor 594 (Thermo Fisher Scientific, United States) and incubated for 2 h, and stained with DAPI (Sigma, United States). All the sections were observed *via* a confocal microscope (LSM 800, ZEISS, Germany). Three independent researchers scored at least 10 fluorescent stains in each group. The positive expression aera in glomerulus was calculated using Image-Pro Plus (Version 6.0, Media Cybernetics, United States).

### Western Blotting

The protein samples were then boiled with a ×5 sample buffer and electrophoresed on a 10% or 12% polyacrylamide gel, then transfer to PVDF membranes. The PVDF membranes were blocked in 5% skim milk for 2 h and incubated overnight at 4°C with rabbit anti-LC3A/B (#12741, CST, United States), rabbit anti-p62 (#16177, CST, United States), rabbit anti-β-catenin (#8480, CST, United States) or rabbit anti-GAPDH (#2118, CST, United States) at 4°C, respectively. The membranes were then washed three times for 10 min in PBS with 0.1% Tween-20 and incubated with anti-rabbit secondary antibody in blocking buffer at room temperature for 1 h. Finally, the membranes were scanned with the Odyssey infrared imaging system (LI-COR Biosciences, NE, United States). All protein bands were analyzed by ImageJ software (Version 1.53, Bethesda, United States).

### Preparation of MFSD and Ultra-Performance Liquid Chromatography/Mass Spectrometry Assay

The ingredients of MFSD were purchased from Guangdong Yifang Pharmaceutical Co., ltd., Foushan, China. Each herb was adjusted to the therapeutic dose of the rat based on the conversion coefficient of the clinical dose of the herb. MFSD was dissolved in water and then stored at 4°C. The contents of MFSD are shown in Table 1.

**TABLE 1:** The composition of MFSD.

TCM	Latin Name	Part Used	Lot. Number	Dry Weight (g) of Daily Clinic Dose
Ma Huang	*Ephedra sinica* Stapf	stem	7051972	20
Fu Zi	*Aconitum carmichaelii* Debx	lateral radix	6120142	20
Gan Jiang	*Zingiber officinale* Rose	rhizome	7090862	30
Fu Ling	*Poria cocos (Schw.)* Wolf	Sclerotium	7010742	30
Bai Zhu	*Atractylodes macrocephala* Koidz	rhizome	6126142	10
Gan Cao	*Glycyrrhizae uralensis* Fisch	rhizome	7021762	10

### Identification of Active Compounds and Related Targets in MFSD

To determine the chemical ingredients of the six herbs in MFSD, we searched the Traditional Chinese Medicine Systems Pharmacology Database (TCMSP, http://old.tcmsp-e.com/tcmsp.php). The absorption, distribution, metabolism, and excretion (OAME) system in this study includes predicting oral bioavailability (OB) and drug-likeness (DL). Meanwhile, compounds were retained only if OB ≥ 30% and DL ≥ 0. 18 to satisfy the criteria suggested by the TCMSP database (Ru et al., 2014). By analyzing the ingredients and target interactions obtained from TCMSP Database, the integrative efficacy of the ingredients in MFSD was determined.

### Prediction of the Therapeutic Targets Acting on MN

MN targets were found in the GeneCards database (https://www.genecards.org/), which contains detailed information on all annotated and predicted human genes.

### Network Construction and Analysis

Interactions between proteins of putative targets of active drugs in MFSD and known therapeutic targets for MN were integrated with putative MFSD target-known therapeutic targets of the MN network to build putative MFSD target-known therapeutic targets of the MN network. It can be used to show the connections between the suspected targets in MFSD and recognized treatment targets. Cytoscape software was used to visualize the graphical interactions in this network (version 3. 6. 0, Boston, MA, United States).

### Protein-Protein Interaction Data

These data of protein-protein interaction (PPI) are from STRING (http://stringdb. org/, version 10), a database of known and forecasted protein-protein interactions (von et al., 2003) with the species limited to “*Homo sapiens*” and the confidence score >0. 7. Each node represents a protein, and each edge represents an interaction. More edges mean more importance of the node.

### Gene Ontology and KEGG Enrichment Analysis for Targets

In order to validate whether potential targets are related to MN, we examined the gene ontology (GO) involving biological process (BP), molecular function (MF), and cellular component (CC). An enrichment analysis according to Tokyo Encyclopedia of Genes and Genomes (KEGG) was conducted to predict the potential signaling pathway of MFSD in MN. The GO and KEGG enrichment analysis was performed using the functional annotation tool of DAVID Bioinformatics Resources 6.7 (https://davidd.ncifcrf.gov/) (Huang et al., 2009). With Benjamini–Hochberg method to control the false discovery rate (FDR) for multiple hypothesis tests, the adjusted *p*-value < 0. 05 was used as the significance cutoff.

### Statistical Analysis

Data were presented as mean ± SEM. One-way analysis of variance was adopted to analyze the statistics *via* a two-sided t-test (two groups) or one-way analysis of variance (ANOVA) followed by Bonferroni’s multiple comparison test (>2 groups). *p* < 0.05 was considered statistically significant.

## Results

### MFSD can Reduce 24 hUTP, CHO, and TG in PHN Rats

**FIGURE 1:**
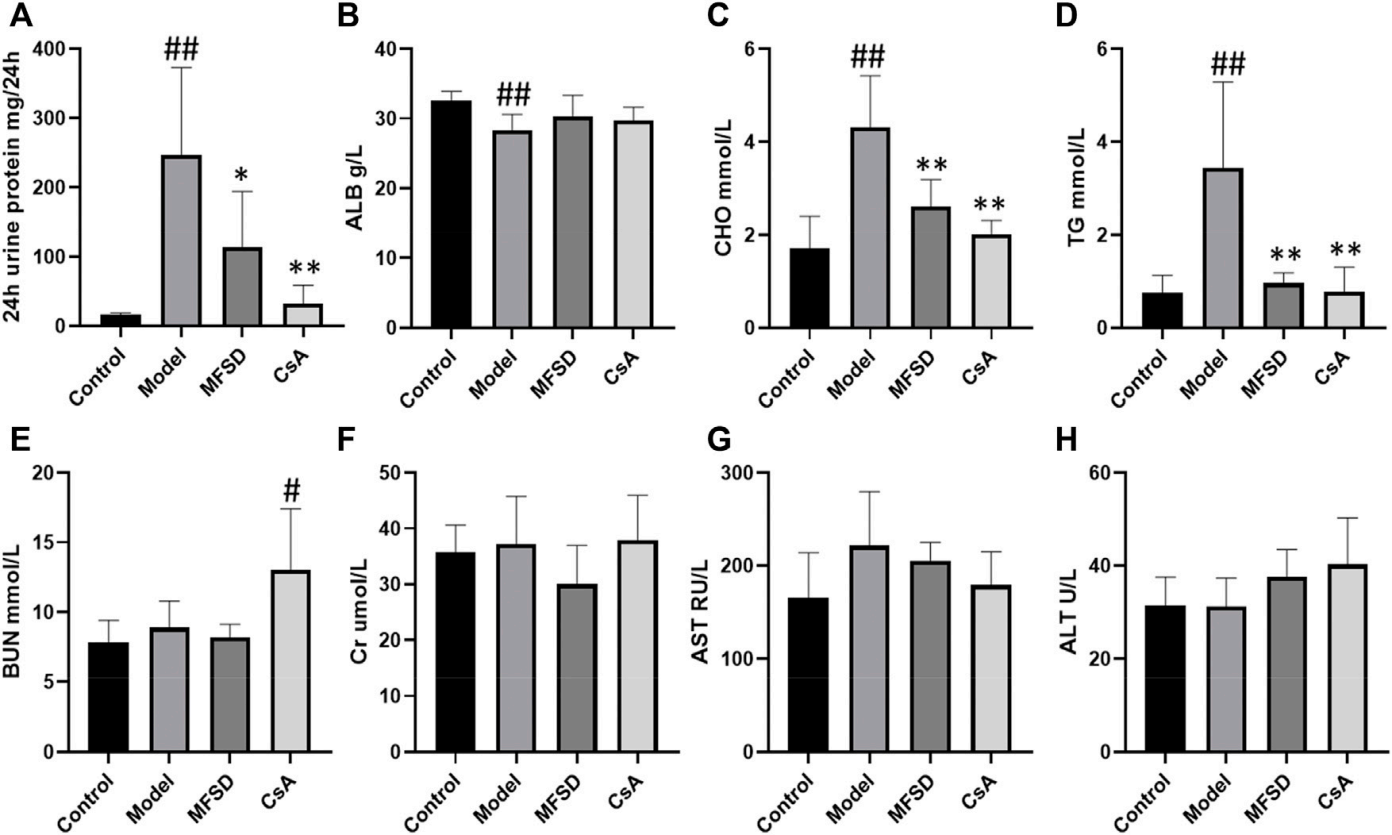
The effect of MFSD on the urine protein and serum biochemical indicators in PHN rats**. (A)** The contents of 24 h urine protein excretion in the last week (*n* = 8) **(B)** The levels of ALB in serum (*n* = 8) **(C, D)** The levels of TC and TG in serum (*n* = 8) **(E, F)** The levels of BUN and Cr in serum (*n* = 8) **(G,H)** The levels of ALT and AST in serum (*n* = 8). Data were expressed as mean ± SD. ^#^ *p* < 0.05 and ^##^ *p* < 0.01 *vs*. control group, *p < 0.05 and **p < 0.01 *vs*. model group.

To evaluate the efficacy of MFSD in PHN rats, we examined the 24 h urinary protein excretion (24 hUTP), albumin (ALB), total cholesterol (CHO), triglycerides (TG), creatinine (Cr), blood urea nitrogen (BUN), alanine aminotransferase (ALT), and aspartate aminotransferase (AST). As shown in Figures 1A,C,D compared with the control group, 24-h urinary protein, CHO, TG significantly increased in PHN rats (*p* < 0.01). Meanwhile, MFSD reduced 24 hUTP after treatment compared with the model group (*p* < 0.05), and CsA reduced the proteinuria excretion (*p* < 0.01). In addition, MFSD and CsA significantly reduced serum levels of CHO and TG (*p* < 0.01). Although there was no statistical significance in ALB between the groups, it can be seen from Figure1B that ALB in the model group was lower than that in the normal group, while MFSD and CsA could improve levels of ALB. At the same time, the BUN, Cr, ALT, AST of the MFSD group were not significantly abnormal compared with the control group, reflecting that MFSD has no obvious hepatotoxicity and renal toxicity in rats (Figures 1E–H).

### MFSD Ameliorates Glomerular Pathomophology and Podocyte Injury in PHN Rats

**FIGURE 2:**
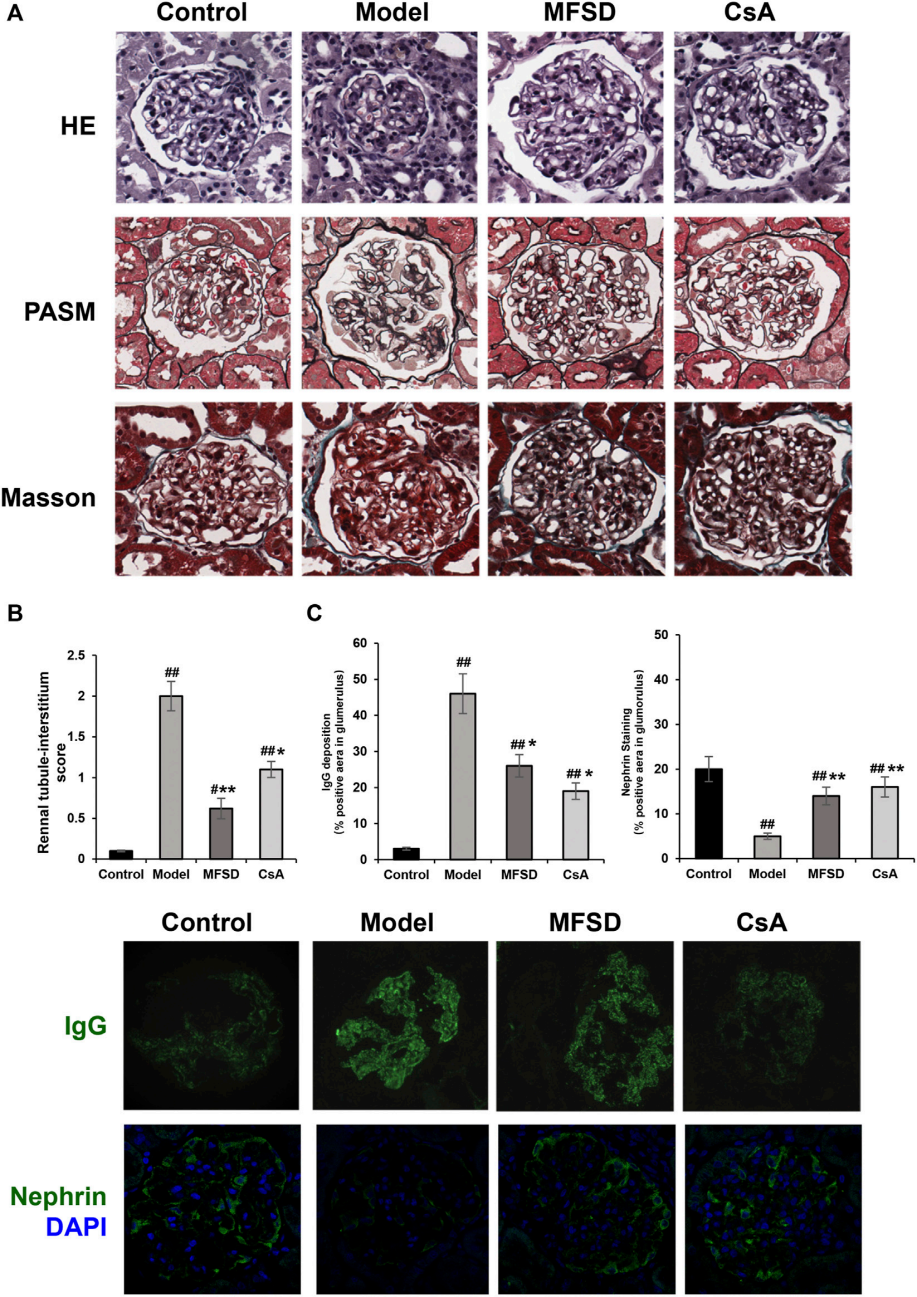
MFSD ameliorated glomerular pathomorphological and podocyte injuries in PHN rats**. (A)** Representative renal pathological staining images of different groups. Top, the images of renal tissues by HE staining were obtained under a light microscope (×400 magnification) (scale bar = 20 μm), Middle and bottom, images of renal tissues by PASM staining and Masson staining were obtained under a light microscope (×400 magnification) (scale bar = 20 μm) **(B)** Histogram is the statistical results of renal tubule-interstitium score of different groups; **(C)** Representative images of IgG and nephrin were observed under a confocal microscope at ×400 magnification (scale bar = 20 μm).

As shown in Figure 2A, the morphological changes of different groups of kidneys were indicated *via* the optical microscope. Diffusion thickening of the basement membrane was observed in the model group. PASM staining showed the formation of “spike process,”, vacuoles and granular degeneration of renal tubular epithelial cells, flattening, exfoliation, and regeneration of epithelial cells, while HE staining showed inflammatory cell infiltration in the renal interstitium (Figure 2B).

In the CsA group, the basement membrane was diffusely thickened, forming the “nailing process”. Renal tubules and interstitial changes were similar to those in the model group. In addition, the appearance of segmental sclerosis in the glomeruli could be noted. MFSD group showed “spike” like changes. We found through renal tubule-interstitium score analysis that the changes in renal tubules and interstitium of MFSD group were reduced compared with the model (*p* < 0.01) and CsA groups (Figure 2A). IgG was expressed in the glomeruli in the model group, while IgG expression in the MFSD and CsA groups was comparatively lower (*p* < 0.05). It can be referred that MFSD and CsA have therapeutic effects on PHN rats, reducing renal injury and immune injury (Figure 2C).

In addition, immunofluorescence showed that the expression of nephrin in glomerulus of model group was significantly down-regulated (*p* < 0.01), while MFSD or CSA treatment could improve the expression of the down-regulated nephrin in glomerulus (*p* < 0.01) (Figure 2C). This result suggests that similar to CsA, MFSD can improve the damage of glomerular podocytes.

### Herbal Compounds in MFSD

**FIGURE 3:**
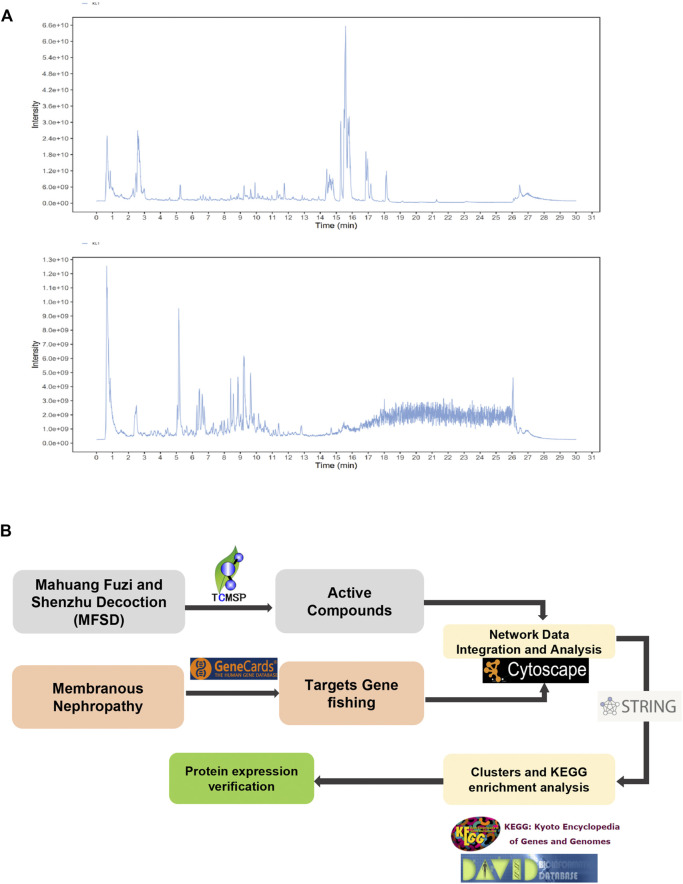
Analysis of the target of MFSD based on network pharmacology **(A)** UPLC-MS Assay of the concentration of MFSD. **(B)** Schematic flow chart of network pharmacology analysis of target of MFSD.

Figure 3A shows a typical chromatogram. According to the TCMSP database, 945 compounds were retrieved, including 363 species of Mahuang, 65 species of Fuzi, 148 species of Ganjiang, 55 species of Baizhu, 34 species of Fuling, and 280 species of Gancao. According to the criteria of OB ≥ 30% and DL ≥ 0.18, 106 chemical ingredients were screened out, among which 23 species of Mahuang, 21 species of Fuzi, 5 species of Ganjiang, 7 species of Baizhu, 15 species of Fuling, and 92 species of Gancao. After taking out the duplicated parts, combined with the results of UPLC-MS assay, 103 chemical constituents were accepted. Finally, 26 chemical ingredients were screened out for further target prediction analysis. (Figure 3B and Table 2).

**TABLE 2:** Compounds in MFSD with oral bioavailability (OB) larger than 30% and drug-likeness (DL) larger than 0.18, which combined with the results of ultra-performance liquid chromatography-mass spectrometry (UPLC-MS).

Comp	Molecule name	OB(%)	DL
1	Herbacetin	36.07	0.27
2	kaempferol	41.88	0.24
3	delphinidin	40.63	0.28
4	quercetin	46.43	0.28
5	Supraene	33.55	0.42
6	naringenin	59.29	0.21
7	Pectolinarigenin	41.17	0.3
8	(+)-Leucocyanidin	37.61	0.27
9	Deoxyandrographolide	56.3	0.31
10	isotalatizidine	50.82	0.73
11	kaempferol	41.88	0.24
12	Isolicoflavonol	45.17	0.42
13	quercetin	46.43	0.28
14	Calycosin	47.75	0.24
15	Medicarpin	49.22	0.34
16	isorhamnetin	49.6	0.31
17	Glabrone	52.51	0.5
18	Glabridin	53.25	0.47
19	naringenin	59.29	0.21
20	liquiritin	65.69	0.74
21	formononetin	69.67	0.21
22	Licochalcone B	76.76	0.19
23	Poricoic acid A	30.61	0.76
24	pachymic acid	33.63	0.81
25	α-Amyrin	39.51	0.76
26	8β-ethoxy atractylenolide Ⅲ	35.95	0.21

### Identification of MFSD-Related Targets

**FIGURE 4:**
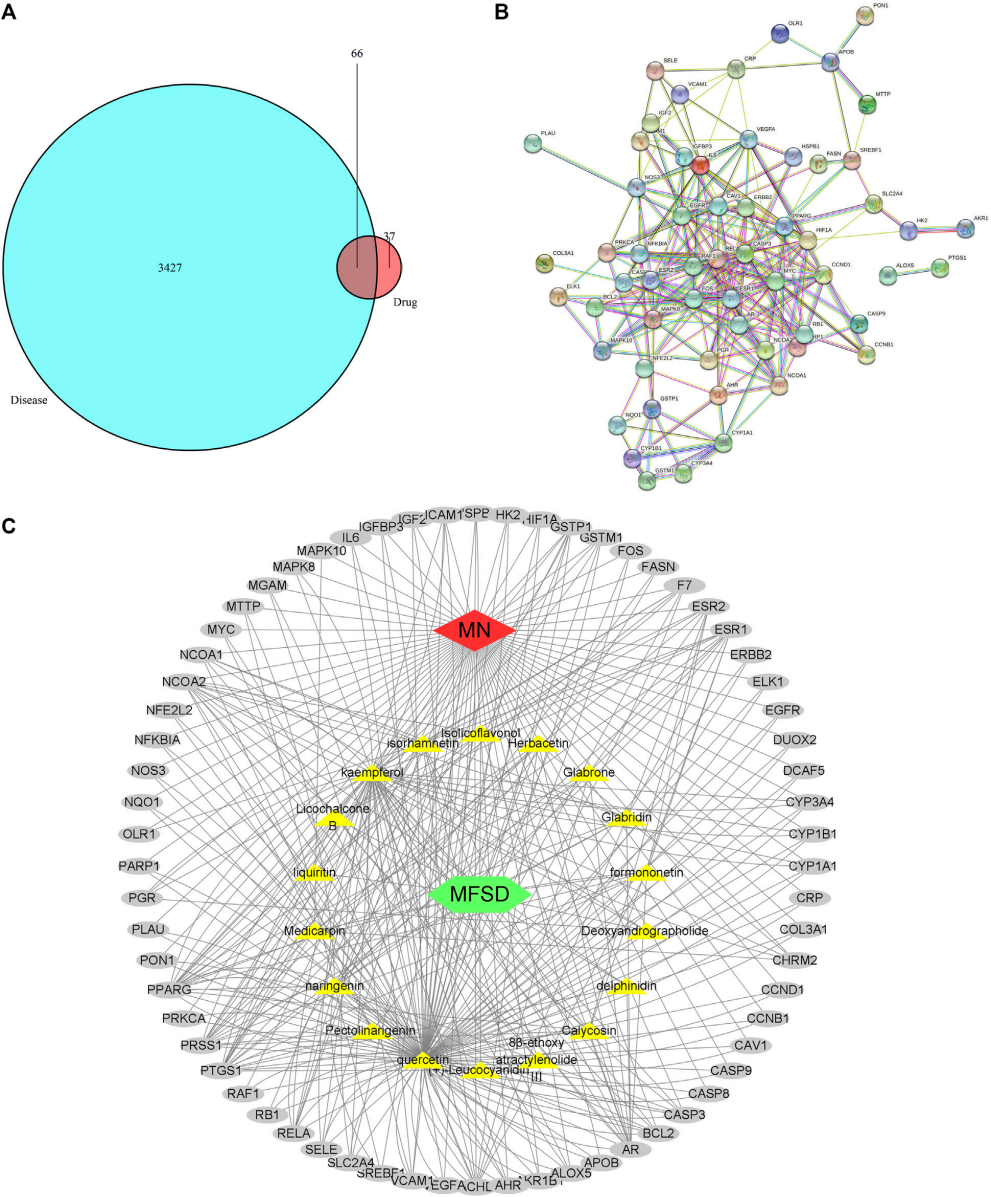
Active compound of MFSD target MN and protein–protein interaction (PPI) network analysis **(A)** Venn diagram of drug-disease targets. The cyan circle represents 3,427 known therapeutic targets for the treatment of MN. The red circle represents 103 known drug targets of MFSD. In total, 66 shared targets were shown between MFSD and MN. **(B)** The PPI analysis of 66 overlapping targets of MFSD and MN. The nodes get larger with an increasing degree. Edges: PPI between shared targets and their interactive partners **(C)** Potential Compound-Targets-Pathway (pC-T-P) network of MFSD in the treatment of MN. There were 6 kinds of herbs, 18 compounds, 66 predicted targets. The yellow hexagon represents 6 kinds of herbs in MFSD, while the grey circle represents potential targets. (For interpretation of the references to color in this figure legend, please refer to the Web version of this article.)

As a result, GenesCard produced 3,427 distinct targets associated with MN, including almost all the targets related to MN that have already been identified or are currently being investigated. Potential targets of MFSD include genes associated with MN progression or treatment. 66 of the 3,427 MN-related genes were closely associated with MFSD. Figure 4A shows the number of overlaps between MFSD-related genes and MN-related genes.

86 nodes and 346 edges (Figures 4B,C) were included in a global view of the compound-target–pathway (C-T-P) network, clarifying the specifics of the MFSD mechanism. As this network demonstrates, MFSD components and their targets are intricately linked. These findings suggested that MFSD interacted with MN in a multi-target, multi-pathway, and overall integrative manner.

### Enrichment Analysis and Construction of the Regulatory Network

**FIGURE 5:**
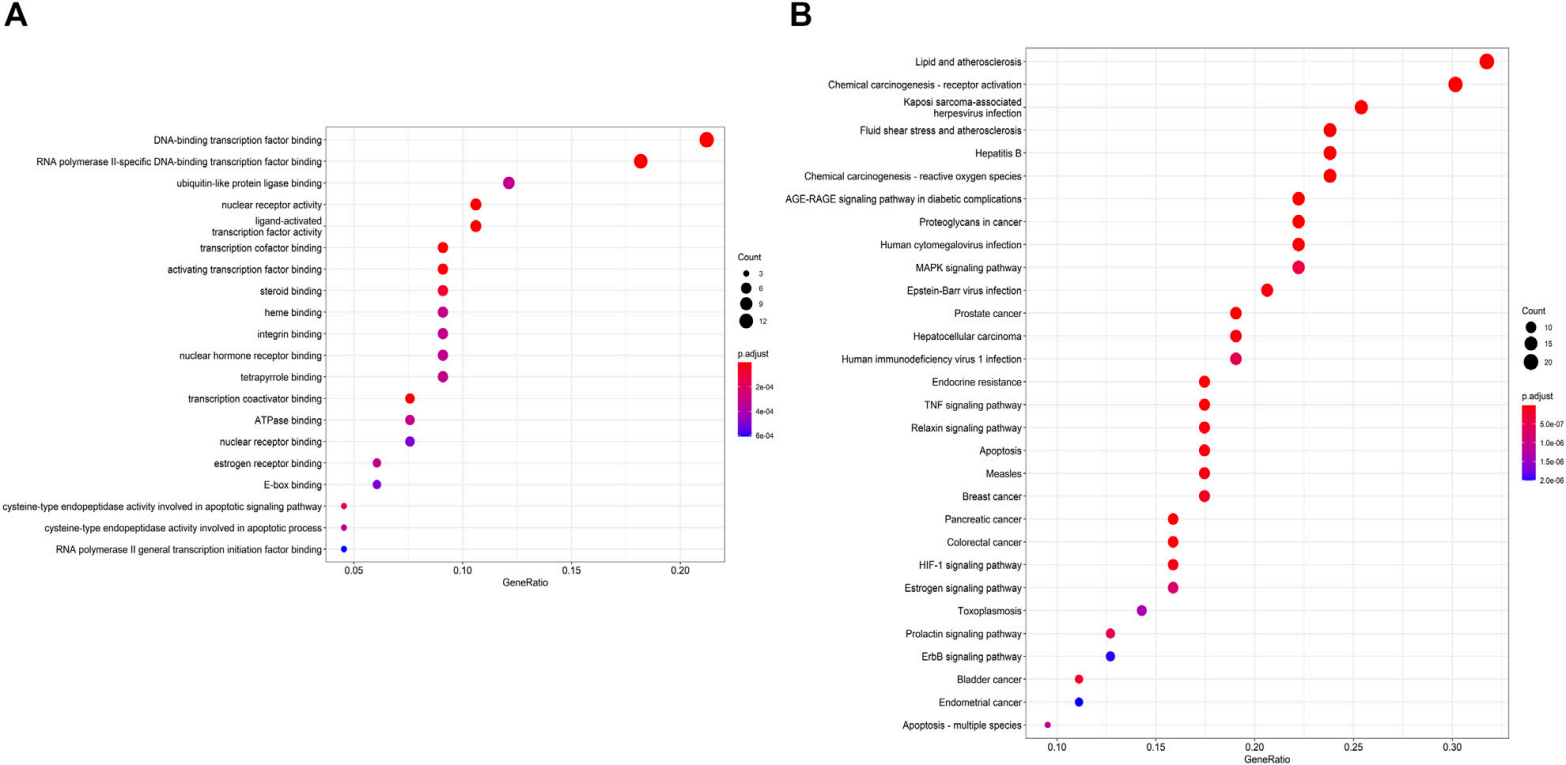
Gene Ontology (GO) enrichment and KEGG pathways analysis for drug-disease targets **(A)** The top 20 significant GO enrichment analyses. The red circle represents the gene count of each biological process. **(B)** The top 30 significant KEGG pathways. (For interpretation of the references to color in this figure legend, please refer to the Web version of this article.)

The above-mentioned possible MFSD target genes were then imported into KEGG pathway enrichment to investigate potential signaling pathways for MFSD in the treatment of MN. Figure 5 shows the top 30 possible signaling pathways. The MAPK signaling pathway (hsa04010), apoptosis (hsa04210), autophagy—animal (hsa04140), Wnt signaling pathway (hsa04310) were discovered to be involved in apoptosis, inflammation, immunity, or oxidative stress biological processes in MN.

### MFSD Adjusted Podocyte Autophagy in PHN Rats

**FIGURE 6:**
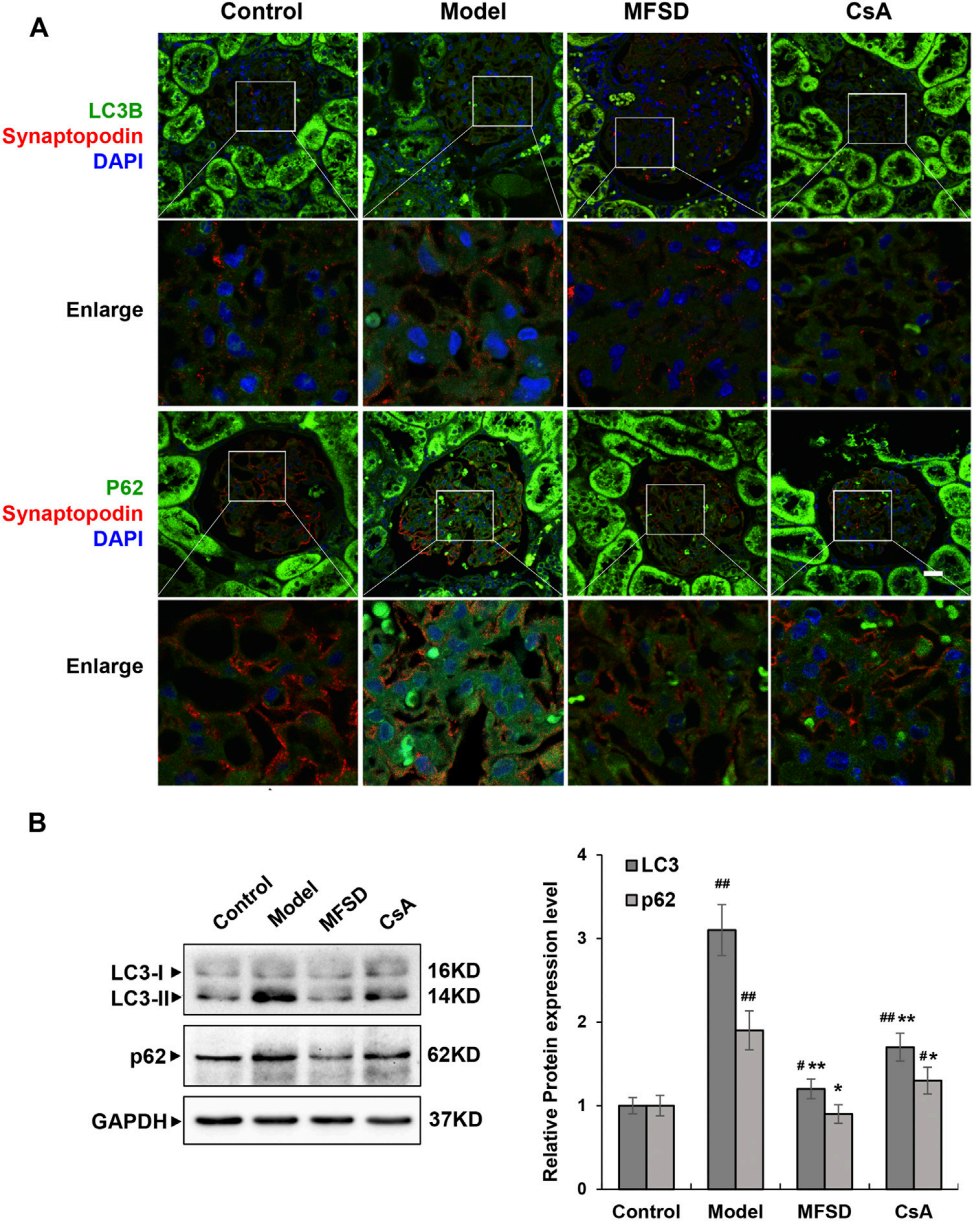
MFSD adjusted podocyte autophagy in PHN rats **(A)** Immunofluorescent analysis of LC3B-positive puncta (green) and p62-positive puncta (green) accumulated in podocytes of PHN. The podocytes in glomeruli were identified by immunofluorescent double-labeling with synaptopodin (red), a podocyte foot process-specific protein. The nuclei were stained with DAPI (blue). Images were obtained using a confocal microscope. **(B)** The relative protein expression levels of renal LC3I/II and p62 of PHN rats were analyzed by western blot assay. The relative protein expression level was expressed as the target protein/GAPDH ratio. Values are represented as mean ± SD. ^#^ *p* < 0.05 and ^##^ *p* < 0.01 *vs*. control group, *p < 0.05 and **p < 0.01 *vs*. model group.

Studies have shown that autophagy is down-regulated in MN (Liu et al., 2017; Jin et al., 2018; Yang et al., 2021). In order to clarify the autophagy in MN, it needs to be explored whether the pathogenesis of PHN involves changes in autophagy and whether the therapeutic effect of MFSD is related to this change. We analyzed the expressions of p62 and LC3B in kidney tissue by immunofluorescence staining (Figure 6A) and Western blot methods (Figure 6B). As shown in Figure 6B, compared with the control group, the expressions of LC3-II and p62 in the model group were higher than that in the control group (*p* < 0.01), whereas the expressions of LC3-II (*p* < 0.01) and p62 (*p* < 0.05) were reduced in the MFSD group, respectively. These data indicated that the treatment of PHN rats by MFSD was at least partially achieved by regulating autophagy.

### MFSD Inhibits the Wnt/β-Catenin Pathway in PHN Rats

**FIGURE 7:**
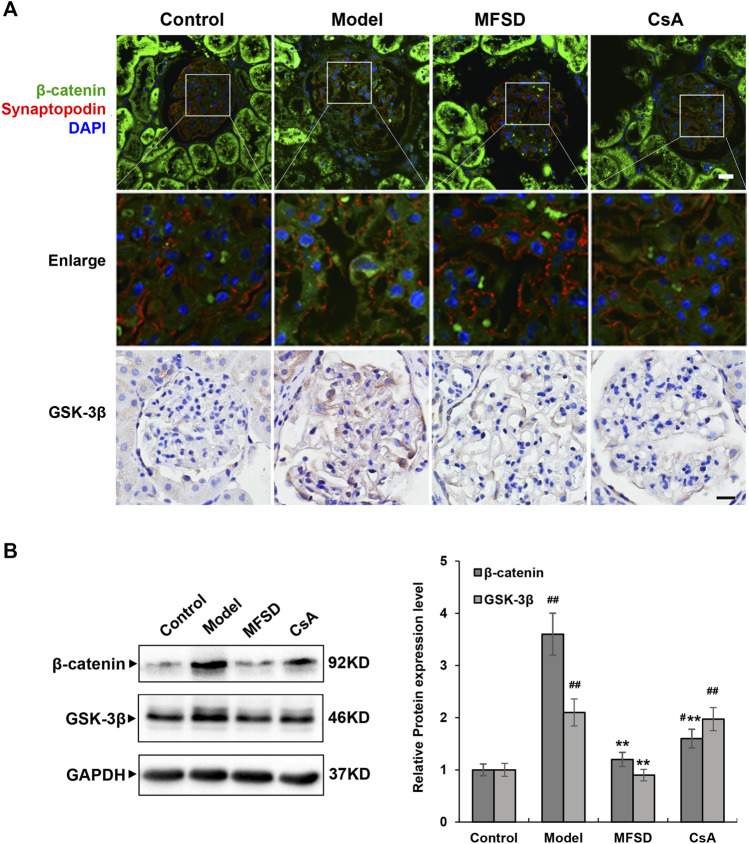
MFSD inhibits the Wnt/β-catenin pathway in PHN rats **(A)** Immunofluorescent staining of β-catenin-positive puncta (green) accumulated in podocytes of PHN. The podocytes in glomeruli were identified by immunofluorescent double-labeling with synaptopodin (red), a podocyte foot process-specific protein. The nuclei were stained with DAPI (blue). Immunohistochemical staining of GSK-3βwas conducted using a confocal microscope. Images were collected under a light microscope at ×400 magnification (scale bar = 20 μm). **(B)** The relative protein expression levels of renal β-catenin and GSK-3β of PHN rats were analyzed by western blot assay. The relative protein expression level was expressed as the target protein/GAPDH ratio. Values are represented as mean ± SD. ^#^ *p* < 0.05 and ^##^ *p* < 0.01 *vs*. control group, **p < 0.01 *vs*. model group.

More studies have shown that the Wnt/β-catenin pathway is critical in CKD pathogenesis. According to cancer-related research, the Wnt/β-catenin pathway is related to autophagy, but the relationship in membranous nephropathy remains unclear. As shown in Figure 7A, immunofluorescence and immunohistochemical staining showed that the model group has a higher expression of β-catenin and GSK-3β compared with the control group (*p* < 0.01). In addition, in the MFSD group, both protein levels of β-catenin and GSK-3β were decreased compared with model group (*p* < 0.01). However, there was only β-catenin in CsA treatment group, but no significant decrease in GSK-3β (Figure 7B). Our results indicate that MFSD may improve podocyte injury by inhibiting Wnt/β-catenin pathway.

## Discussion

Idiopathic membranous nephropathy is a common cause of adult nephrotic syndrome (Ponticelli and Glassock, 2014). In clinical practice, about 70% of patients present with nephrotic syndrome, and 30% of patients can spontaneously relieve, but patients with large proteinuria will develop into end-stage renal disease (Polanco et al., 2010; Moroni et al., 2017). The current medications for the MN treatment are mainly cyclosporine, cyclophosphamide, and rituximab, but serious side effects still exist (van den Brand et al., 2017; Zhang et al., 2018).

MFSD is a common clinical prescription to treat kidney disease. It is mainly composed of ephedra, aconite, licorice, dried ginger, poria, and fried atractylodes, and its main components have been identified as effective. Moreover, traditional Chinese medicine prescriptions other than a single ingredient have effectively treated various diseases for hundreds of years. Our team has found that MFSD is effective in treating MN through clinical applications, but the specific mechanism is still unknown. The classic animal model of MN is intravenously injected with anti-Fx1A Serum named Passive Heymann Nephritis (Heymann et al., 1959). Therefore, in our study, a PHN rat model induced by Fx1A injection was performed to evaluate the effect of MFSD on MN.

The “one-drug and one target” mode is not appropriate to develop TCM’s action mechanism because it is characterized by a multi-component, multi-target, and multi-pathway synergistic action mode. Network pharmacology is a new approach that combines system biology, multi-directional pharmacology, and computational biology to investigate the interaction between medications and diseases from a broad perspective. It is particularly well suited to elucidating the complicated interaction between medications, targets, pathways, and illnesses. In this study, a global perspective of the prospective chemical target pathway network was established using network pharmacology to investigate the molecular mechanism and potential targets of MFSD in MN treatment.

The active components related to MFSD were obtained from TCMSP. Through the enrichment of biological pathways in the Mendelian genetic and KEGG database, the protein targets in MFSD were screened. In addition to active components and target genes, several meaningful signal pathways include apoptosis, autophagy, the TNF, MAPK, and Wnt/β-catenin pathways. Abundant biological functions and literature studies suggest that these pathways are mainly related to oxidative stress, apoptosis, inflammation, and immune response involved in the progression of MN, providing a preliminary understanding and stimulating the interest in further research.

After preliminarily determining the role of the multidimensional regulatory network in MN treatment, MFSD was verified in the PHN rat model. PHN rats treated with MFSD can significantly reduce proteinuria, slightly increase serum albumin without obvious liver and kidney function abnormalities. Therefore, it can be concluded that MFSD has a potential therapeutic effect on MN.

There are 19 different secreted proteins in Wnts. The Wnt/β-catenin signal cascade plays an important role in organogenesis, tissue homeostasis, and human diseases (MacDonald et al., 2009; Clevers and Nussw, 2012). Wnt/β-catenin signaling pathway-related proteins are expressed in the kidney, especially in tubular epithelial cells, fibroblasts, and macrophages (Schunk et al., 2021). Evidence has been accumulated that the Wnt/β-catenin signaling pathway is closely related to kidney disease, and relevant research is mostly about inflammation and fibrosis (Tan et al., 2014; Edeling et al., 2016). Regarding the Wnt/β-catenin signaling pathway and the occurrence of proteinuria, it is found that β-catenin can inhibit the expression of nephrin, leading to the destruction of the glomerular slit diaphragm and the formation of proteinuria (Dai et al., 2009). MN manifests as podocyte damage in the kidney, but the mechanism of protective action of MFSD in MN is unknown. In our study, PHN rats were observed to activate the Wnt/β-catenin signaling pathway compared with the control group. Interestingly, we discovered that in the model group, β-catenin levels increased considerably, indicating aberrant Wnt/β-catenin signaling. The expression of these Wnt/β-catenin signaling-related proteins was considerably reduced in the MFSD groups, showing that MFSD prevented the Wnt/β-catenin signaling pathway from becoming overactive.

However, it is not sufficient to explain the remission of proteinuria in MN patients with MFSD, and further research is needed to rule out the entire mechanism. Although it was documented that autophagy could be inhibited in MN, our experimental results showed that the expression levels of autophagy-related proteins p62 and LC3B increased in the model group but decreased in the MFSD group, which meant that autophagy was inhibited in the MFSD group.

Autophagy, being highly conservative, is a common living phenomenon in eukaryotic cell organisms. It is a programmed cell death mechanism alongside apoptosis and necrosis. Autophagy plays an extremely important role in cell waste removal, structural reconstruction, organelle renewal, and growth and development (Levine and Kroemer, 2008; Kroemer et al., 2010). Some intracellular contents degrade under physiological and pathological conditions (Kroemer, 2015; Gatica et al., 2018; Wong et al., 2018). Podocytes are cells that maintain high levels of autophagy (Bork et al., 2020). At the same time, abnormal autophagy exists in chronic kidney disease (Liu et al., 2017; Jin et al., 2018; Zhou et al., 2019; Li et al., 2021; Yang et al., 2021). Studies in membranous nephropathy have shown that autophagy is down-regulated in membranous nephropathy (Liu et al., 2017; Yang et al., 2021), but the mechanism leading to its down-regulation is not yet clear. Our experimental results show that autophagy protein expression in the PHN rat model group tends to increase, which is statistically significant.

To clarify the situation of autophagy in MN, our team believes that the mechanism of inhibiting/activating autophagy is related to the Wnt pathway. The main regulator of autophagy is the mTOR pathway (Ravikumar et al., 2004; Kim et al., 2011). Some studies have also shown the complex relationship between autophagy and the Wnt/β-catenin pathway (Petherick et al., 2013; Kühn et al., 2015; Yun et al., 2020; Zhou et al., 2021). Autophagy and Wnt/β-catenin pathway-related proteins are highly expressed in cancer cells (Zhou et al., 2019), and the Wnt/β-catenin pathway is a negative regulator of p62 (Petherick et al., 2013). However, there is no research on this pathway in MN.

Meanwhile, our research discovered that inhibiting the Wnt/-catenin signaling pathway lowered autophagy in complement-treated podocytes in cellular experiments (Dong et al., 2021). Our study showed that in MN, the expression of the Wnt/β-catenin pathway and autophagy in podocytes were up-regulated, while the expression of Wnt/β-catenin and autophagy were down-regulated after the application of MFSD. This study confirmed the connection between the Wnt/β-catenin signal and autophagy in MN, also proving that MFSD could treat MN through this pathway.

MFSD is a traditional Chinese medicine compound that treats membranous nephropathy through multiple targets, and one of the mechanisms may be to inhibit the Wnt/β-catenin signaling pathway and autophagy. The main therapeutic ingredient of MFSD may be ephedrine, ephedra polysaccharide, or other effective ingredients, which our team will further explore. At the same time, we will also explore more targets for MFSD to treat MN.

## Data Availability

The original contributions presented in the study are included in the article/Supplementary Material, further inquiries can be directed to the corresponding authors.
